# Explaining Lifelong Loyalty: The Role of Identity Fusion and Self-Shaping Group Events

**DOI:** 10.1371/journal.pone.0160427

**Published:** 2016-08-10

**Authors:** Martha Newson, Michael Buhrmester, Harvey Whitehouse

**Affiliations:** Institute of Cognitive and Evolutionary Anthropology, University of Oxford, Oxford, Oxfordshire, United Kingdom; University of Westminster, UNITED KINGDOM

## Abstract

Pledging lifelong loyalty to an ingroup can have far-reaching behavioural effects, ranging from ordinary acts of ingroup kindness to extraordinary acts of self-sacrifice. What motivates this important form of group commitment? Here, we propose one especially potent answer to this question–the experience of a visceral sense of oneness with a group (i.e., identity fusion). In a sample of British football fans, a population in which high levels of lifelong loyalty are thought to be common, we first examined the hypothesised relationship between fusion and perceptions of lifelong loyalty to one’s club. We further explored the hypothesis that fusion and lifelong loyalty are not merely a reflection of past time investment in a group, but also reflect a deeper, memory-based process of feeling personally shaped by key group events, both euphoric and dysphoric. We found broad support for these hypotheses. Results suggest that feeling personally self-shaped by club events (e.g., crucial wins and losses), rather than time invested in the club, leads to greater identity fusion to one’s club. In turn, fusion engenders a sense of lifelong club loyalty. We discuss our findings in relation to the growing literature on the experiential origins of intense social cohesion.

## Introduction

Ingroup loyalty–faithfully sticking with one’s ingroup through thick and thin–courts reciprocal commitment from other group members, thus making group behaviour more predictable and stable [1, 2]. Declarations of loyalty to one’s group are common cross-culturally. For instance, through prayer, hymn or bumper sticker, religious believers around the world express their unwavering commitment to serve their God, Prophet, or other supernatural agent. In sport too, fans travel long distances, devote large amounts of money and time to following and participating in their teams’ events, and often display visual symbols of their allegiance to their team; lifelong and painful tattoos are not uncommon. In terms of public policy, by identifying the mechanisms through which group loyalty develops, we are better placed to harness it for practical and positive outcomes, e.g. acts of charity or the reduction of inter-group violence.

The social identity perspective offers one account of group loyalty. Social identity refers to a person’s sense of who they are in terms of group membership [3]. If an individual is highly identified they will primarily see themselves as a group member, as opposed to a unique individual while low identifiers would see themselves as the latter. When a person’s group membership is active and their sense of self revolves around their group, we would intuitively expect their group loyalty to be relatively high. Indeed, when given the option to move from a low status to a high status group, high identifiers choose to stay with their group while low identifiers do not, regardless of the threat to their identity [4].

However, high identification with one’s group may not be the only route to group loyalty. Recent research has delineated a complementary and potentially more behaviourally potent mode of ingroup cohesion known as 'identity fusion’ [5–7]. Whereas high identifiers report strong adherence to the group category (e.g., group values, norms, etc.), strongly fused persons report a visceral sense of oneness between their personal self and their group identity [8, 9]. Additionally, whereas group interaction activates only the social identities of high identifiers, for fused persons, both the social and personal self are simultaneously activated (6). Recent research has shown that strongly fused as compared to highly identified persons are especially likely to engage in a variety of personally costly, pro-group behaviours [5, 7, 10–12].

Strongly fused persons, emboldened by a sense of personal agency, invulnerability, and sense of familial ties to group members, are willing to put their lives at risk to save ingroup members [9, 11, 13, 14] and provide financial and socio-emotional support to needy ingroup members [9, 15]. Strongly fused group members in longitudinal studies also tend to remain strongly fused over time [6, 16]. Together, this body of evidence suggests that strongly fused persons may exhibit extremely high levels of group loyalty beyond that generated by group identification.

Here, we explore one particularly important form of ingroup loyalty–lifelong loyalty. Lifelong loyalty refers to a willingness to stick with one’s group in perpetuity, remaining faithful to the group through good times and bad and therefore forgo opportunities to abandon the group in favour of more attractive ones [4, 17, 18]. Several studies have assessed the ‘exit problems’ group members face in terms of choosing whether to stick with one’s group or switch to a more appealing scenario (e.g., [4, 18]. In addition to Ellemers et al.’s original work on high identifiers ‘sticking together’ [4], Van Vugt and Hart (17) observe that high identifiers choose to stay when this benefits the group, despite the personal costs involved. To replicate and extend this association to the domain of football fandom, we first sought to test whether those most strongly identified with the ingroup report the highest levels of lifelong club loyalty.

Fusion is likely to be an especially strong predictor of this form of loyalty because for fused individuals, to be unfaithful to the group would be to betray not only the group itself (associated with the sanctity of familial ties) but also the essence of their personal self [14, 15, 19]. For a strongly fused person, to renounce one’s group membership would be tantamount to total rejection of one’s present and past self, an epistemic and practical nightmare. We thus extend previous work by testing whether fusion is associated with lifelong club loyalty, while controlling for group identification.

If fusion engenders lifelong group loyalty, what triggers fusion? The answer may lie in a framework recently developed by Whitehouse and Lanman [20]. This framework proposes that particularly intense life events hold the power to shape the personal autobiographical self [21]. When such life events are shared with ingroup members, through a process of reflection over time, people may in turn perceive that these events were significantly identity-shaping, both personally and socially. As a result, one’s personal and social identities become more closely aligned or fused [7, 22, 23]. Several recent studies have found broad support for this account, however, none have specifically examined linkages between fusion and perceptions of how group events have been personally self-shaping [15, 24, 25]. Therefore in our current study we assessed the extent to which sharing self-shaping experiences with others leads to fusion with the ingroup.

Another key prediction originating from Whitehouse and Lanman [20, 26] is that highly dysphoric (intensely negative) group experiences can affect fusion in ways equal to and even beyond euphoric group experiences. While the power of euphoric events to shape individuals [27, 28] and increase group cohesion [27] is well documented, little research has assessed the effects of dysphoric experiences on perceptions of self-shaping and group bonding, and findings have been mixed. For instance, Turner et al. [29] found increased cohesion following failure or defeat in cooperative tasks and intergroup competitions, but only under conditions of high choice about doing the task or high commitment to the group respectively. This effect was revealed using a minimal groups paradigm run with undergraduate participants and 13–14 year old schoolgirls, so generalisability to longstanding groups, such as football clubs, is unknown. In other work, two meta-analyses have identified associations between positive team performance and group cohesion [30, 31], but interpreting the strength and causal direction of such effects proved difficult because of conceptual and methodological heterogeneity across the many studies analysed [32]. Given this state of affairs, in our study we surveyed participants’ perceptions of feeling self-shaped by both positive and negative group events, examining each separately and together.

In addition to testing for affirmative support for hypotheses derived from Whitehouse and Lanman (19), we also sought to examine a potentially more parsimonious alternative explanation for the development of fusion and ingroup loyalty: cognitive dissonance [17, 33]. Group members who have invested significant time, energy, and resources into their group may be most apt to bring their self-perceptions (group loyalty) into line with their past pattern of behaviour (duration of support), reflecting an effort to reduce cognitive dissonance [34]. In Van Vugt and Hart’s study [17], positive group perception explained group loyalty better than ‘self-perception’ or cognitive dissonance theory. Using relatively minimal groups (e.g., university affiliates) in a laboratory setting, high identifiers’ group loyalty was not explained by a justification of their past investments. Rather, past investment affected group loyalty independently of the effect of identification, suggesting that dissonance theory and social identity form two independent routes to group loyalty. Turner et al.’s study described above [29] also found evidence for a dissonance explanation of increased cohesion following negative events in their laboratory study. We sought to test whether cognitive dissonance results in in-group loyalty in a cohort of real-life, self-identifying group members by returning to Ellemers et al.’s [4] original anecdote on the loyalty of sports fans. Namely, we examined whether past investments (i.e., one’s time supporting a sports team) predicted fusion and self-rated group loyalty for fans of British football teams.

## Current Study

The present study tests three hypotheses. Our first hypothesis is that while identification, past investment, and fusion all predict loyalty when individual relationships are examined, only fusion and past investment predict loyalty when entered into a regression simultaneously. Second, we predict that perceptions of self-shaping events affect loyalty via fusion rather than via past investments (i.e., a statistical mediation hypothesis). Third, we predict that perceptions of self-shaping events following positive (euphoric) *or* negative (dysphoric) events both predict fusion to club.

## Methods

We focused on a group domain central to modern life for billions of humans across the globe–football (or soccer) fandom [35]. We targeted on fans of the UK’s top two football leagues and released the study for a two-week period during a relatively quiet time in the football season (23^rd^ October– 2^nd^ November 2015). Ethical approval was obtained from the School of Anthropology and Museum Ethnography Research Ethics Committee (SAME REC) in accordance with the procedures laid down by the University for ethical approval of all research involving human participants. Participants provided electronic informed consent by checking boxes on a screen in a similar fashion to check boxes on a printed document. Consent was recorded in the survey software and participants could not proceed to the main survey without reading and checking the consent pages.

A short online questionnaire (*N* = 140) was advertised to a diverse cross-section of football fans through social media (Twitter and Facebook), online football forums, and fan blogs, as well as via student and subject-pool mailing lists. Participants could win one of three £100 prizes for completing the survey. To reduce the likelihood of participants rushing the online survey, we informed them of the estimated time required to complete it. We expected that most participants would prefer to complete a brief survey, thus we chose when possible to use abbreviated measures.

Of the 146 participants (*M*_age_ = 37.14, SD = 13.09, range = 18–76), 80.82% were male (15.07% female), 9.6% left education at or before the age of 16, 23.3% had college education (16–18 years), 41.1% had undergraduate education, and 22.6% had postgraduate education. The male skew is broadly representative of gender imbalances in wider football fan demographics and was thus considered relevant to our target population. 94.3% of our sample spoke English as a first language.

### Measures

Identity fusion was assessed using the 7-point verbal scale [7] regarding the individual’s preferred club (*α* = .89). Example items include ‘*I am one with [my club]*, *‘I have a deep emotional bond with [my club]’*, ‘*I’d do more for [my club] than any other fan would’* and *‘[My club] makes me strong’*.

Identification was assessed using the 7-point single item measure [36]: ‘*I identify with [my club]*’.

Ingroup loyalty was assessed via six questions using a 7-point response scale. Items were generated based on definitions of ingroup loyalty, and survey questions from Van Vugt & Hart (2004). The six items below were included regarding the individual’s preferred team. The questions were reverse scored so that high scores reflected high loyalty. The scale was internally consistent (*α* = .82).

1. *How likely is it that you will remain a fan of [club] for the rest of your life*? *(Extremely likely* vs. *Extremely unlikely)*
2. *‘Even though I may not want to now*, *I will probably end up switching to a different team’ (Extremely unlikely* vs. *Extremely likely)*
3. *‘I will never stop supporting [club]’* vs. *‘I won’t support [club] for long’*
4. *‘Nothing could stop me being a fan of [club]’* vs. *‘Nothing could keep me a fan of [club] forever’*
5. *‘When [club] has just lost*, *I…‘…am as strong a fan as ever’* vs. *‘…come close to switching teams’*
6. *‘I could never stop being a fan of my team’* vs. *‘I don’t mind which team I support’*

*Self-shaping club events* were assessed via two questions using a 7-point response scale (*‘Not at all…Extremely’)*. One question asked about euphoric events: *‘To what extent have your team’s wins shaped you as a person*?*’* The second question asked about dysphoric events: ‘*To what extent have your team’s losses shaped you as a person*?*’*. We then calculated a mean ‘self-shapingness’ score. The score composed of the two items was internally consistent, *α* = .82

*Duration of support* was used as a measure of past investment and thus the amount of experienced dissonance. Participants estimated how many years they had supported their team for. We calculated a single variable by making raw years of supporting the team as a percentage of the participant’s age. As our sample included a broad range of ages (18–76) it was sensible to view years of support in relation to the individual’s potential number of years of support. We repeated our analyses using both the age and raw years of support variables and these analyses were similar to those using the single ‘duration of support’ variable, though years of support had a better predictive value than age (Supporting Information 2). Descriptives and correlations for all variables are reported in Table 1.

**Table 1:** Descriptive statistics and correlations for identity fusion, identification, loyalty, self-shaping events, and past investment.

	Self-shap.	Identific.	Investment	Fusion	Loyalty
Self-shap.	3.67 (1.68)				
Identific.	.13 (.11)	5.17 (1.52)			
Investment	.19 (.04)	-.003 (.97)	68.81 (24.72)		
Fusion	.40 (< .01)	.57 (< .01)	.09 (.28)	3.88 (1.38)	
Loyalty	.34 (< .01)	.16 (.06)	.44 (< .01)	.32 (< .01)	6.48 (.73)

Note: Means and SD (in parentheses) on the diagonal, Pearson’s r’s and p-values (in parentheses) below the diagonal.

## Results

### Hypothesis 1: Identification, dissonance, and fusion predict loyalty, but only the effects of dissonance and fusion hold in a simultaneous regression

We ran a linear regression with loyalty as the dependent variable and identification entered in block 1 (*R*^*2*^ = .03, *F(139)* = 3.75, *B* = .16, *p* = .06), investment entered in block 2 (*R*^*2*^ = .21, *F*(138) = 19.11, *B =* .44, *p* < .01), and fusion entered in block 3 (*R*^*2*^ = .27, *F*(137) = 16.96, *B =* .28, *p* < .01) (see Table 2). Consistent with Van Vugt and Hart (17), identification alone in block 1 predicted loyalty. With past investment added to the model in block 2, both variables independently predicted loyalty. However, with fusion added to the model in block 3, the effect of identification was no longer statistically significant while the effect of fusion on loyalty was statistically significant. Overall, our hypothesis was supported. Our model suggests that loyalty may be a result of past investments (dissonance), but a unique pathway to loyalty unexplained by dissonance is fusion.

**Table 2:** Linear regression with identification, past investment, and fusion entered as variables to predict group loyalty.

Model		Unstandardised Coefficients		Standardised Coefficients	t	Sig. (*p*)
		B	Sth. Error	Beta		
1	(constant)	6.07	.22		27.56	< .01
	Identification	.08	.04	.16	1.94	.06
2	(constant)	5.21	.25		20.97	< .01
	Identification	.08	.03	.16	2.12	.04
	Past Investment	.01	.002	.44	5.80	< .01
3	(constant)	5.08	.24		20.86	< .01
	Identification	.001	.04	.002	.02	.98
	Past Investment	.01	.00	.41	5.59	< .01
	Fusion	.15	.05	.28	3.19	< .01

Dependent Variable: Group loyalty

### Hypothesis 2: The path to fusion is self-shapingness, not dissonance

**Fig 1:**
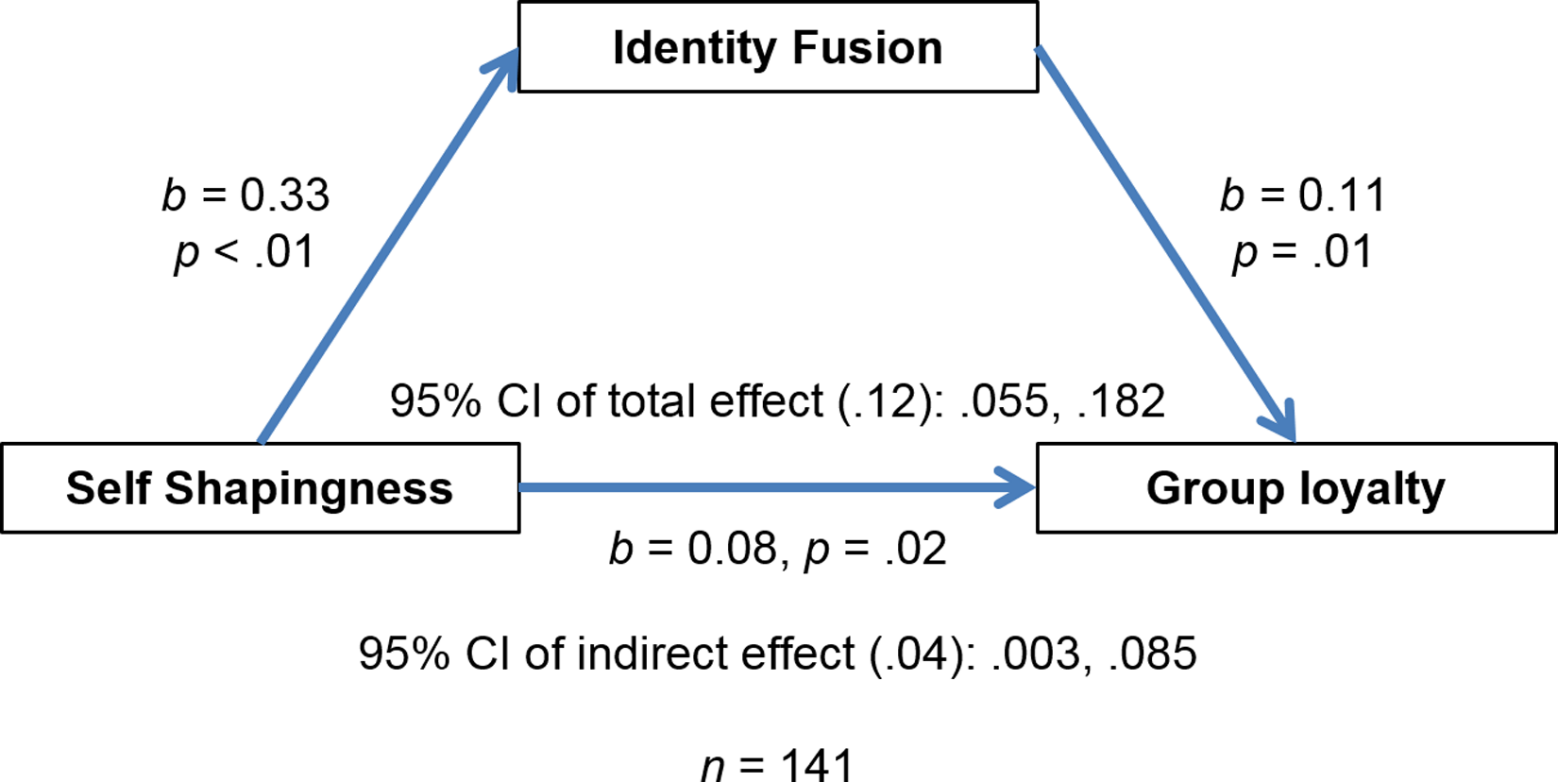
Mediation analysis shows that identify fusion mediates the relationship between self-shapingness and group loyalty.

A simple mediation analysis was conducted using ordinary least squares path analysis in Hayes’s PROCESS macro (Model 4) for SPSS (Hayes, 2013). Bias-corrected bootstrap analyses based on 5,000 bootstrap samples were run. In the model the outcome was loyalty, fusion the mediator, self-shapingness the predictor, and past investment a covariate (Fig 1; past investment not pictured in the figures). As seen in Table 3, the confidence intervals for the indirect effect did not cross zero. There was also evidence of a direct effect of self-shapingness on loyalty, independent of past investment and fusion. We also found that self-shapingness predicted fusion, while past investment did not in a linear regression generated by PROCESS 4, *R*^*2*^ = .16, *F*(1,38) = 13.24, *B* = .33, *p <* .01. (Table 4). In sum, the model supported the hypothesis that dissonance does not mediate the path to fusion and that self-shapingness is an independent source of fusion. We re-ran the model controlling for identification (entered as a covariate) and the results remained relatively robust (Sobel *z* = 1.87, p = .06). We also re-ran the model replacing fusion with identification as the mediator and found the model was not supported (*z* = -2.30, *p* = .82) due to the null relationship between identification and self-shapingness.

**Table 3:** Total, direct, and indirect effects for self-shapingness predicting loyalty (outcome) via fusion and past investment as a covariate.

	Effect	SE	95% CI
Total effect	.12	.03	.055, .182
Direct effect	.08	.03	.015, .150
Indirect effect	.04	.02	.003, .084

**Table 4:** Self-shapingness and past investment as predictors of fusion (outcome) in a linear regression.

	Coefficient	*t*	*p*
Self-shapingness	.33	5.00	< .01
Past investment	.002	0.33	.74

### Hypothesis 3: Self-shapingness following both euphoric and dysphoric events predicts fusion

**Fig 2:**
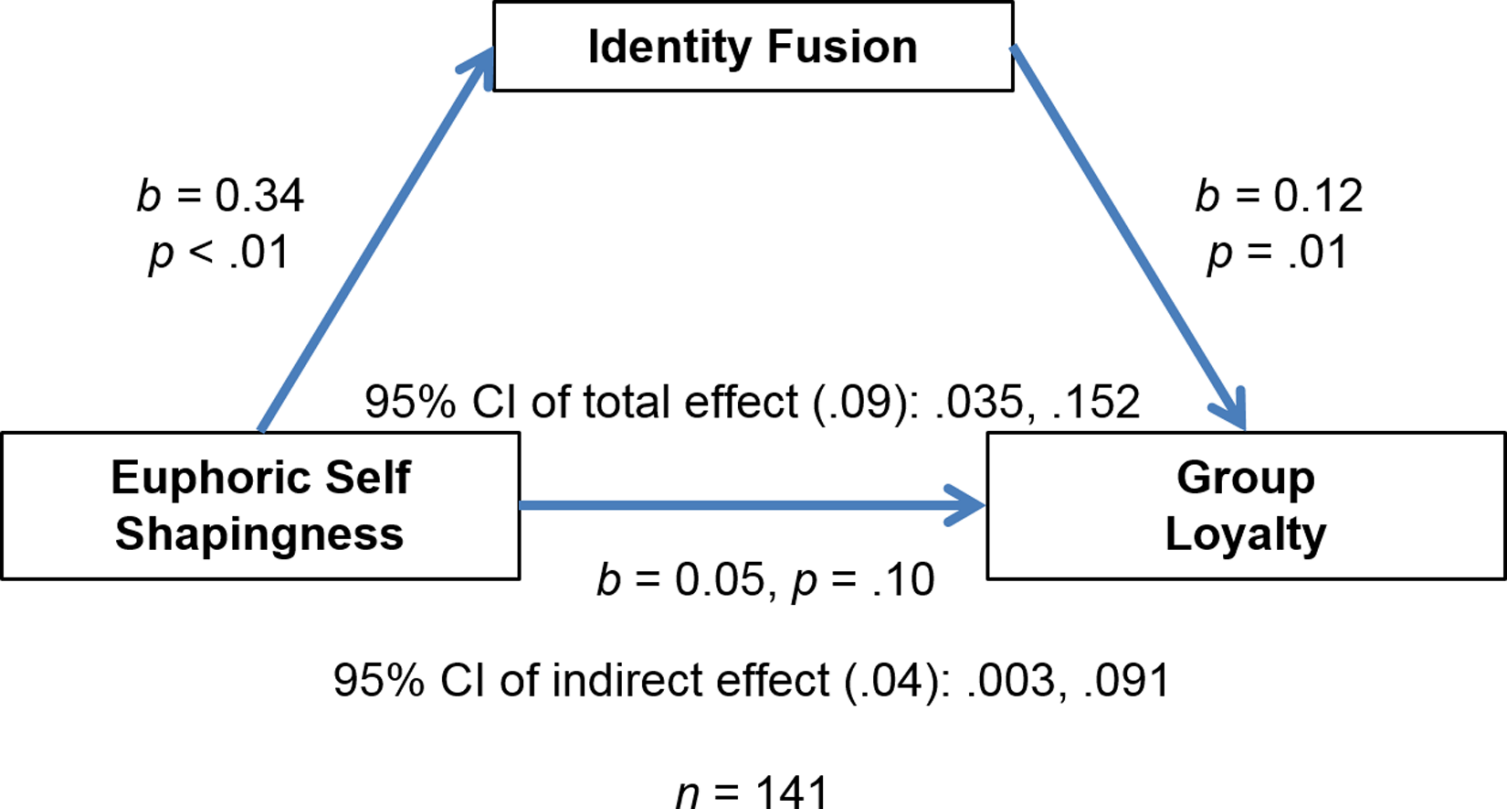
Mediation analysis shows that identity fusion mediates the relationship between euphoric self-shapingness and group loyalty (outcome).

The above model was repeated twice more, but instead of including mean self-shapingness we included self-shapingness for euphoric and dysphoric events separately. In model 1 the outcome was loyalty, fusion the mediator, euphoric self-shapingness the predictor, and past investment a covariate (Fig 2). As seen in Table 5, the confidence intervals for the indirect effects did not cross zero. There was a marginal direct effect in this model. We also found that euphoric self-shapingness significantly predicted fusion, independent of past investment in a linear regression generated by PROCESS Model 4, *R*^*2*^ = .21, *F*(138) = 18.16, *B* = .34, *p* < .01 (Table 6).

**Table 5:** Total, direct, and indirect effects for euphoric self-shapingness predicting loyalty (outcome) with fusion as a predictor and past investment as a covariate.

	Effect	SE	95% CI
Total effect	.09	.03	.035, .152
Direct effect	.05	.03	-.011, .117
Indirect effect	.04	.02	.003, .091

**Table 6:** Euphoric self-shapingness and past investment as predictors of fusion (outcome) in a linear regression.

	Coefficient	*t*	*p*
Self-shapingness	.34	5.89	< .01
Past investment	.001	0.16	.87

**Fig 3:**
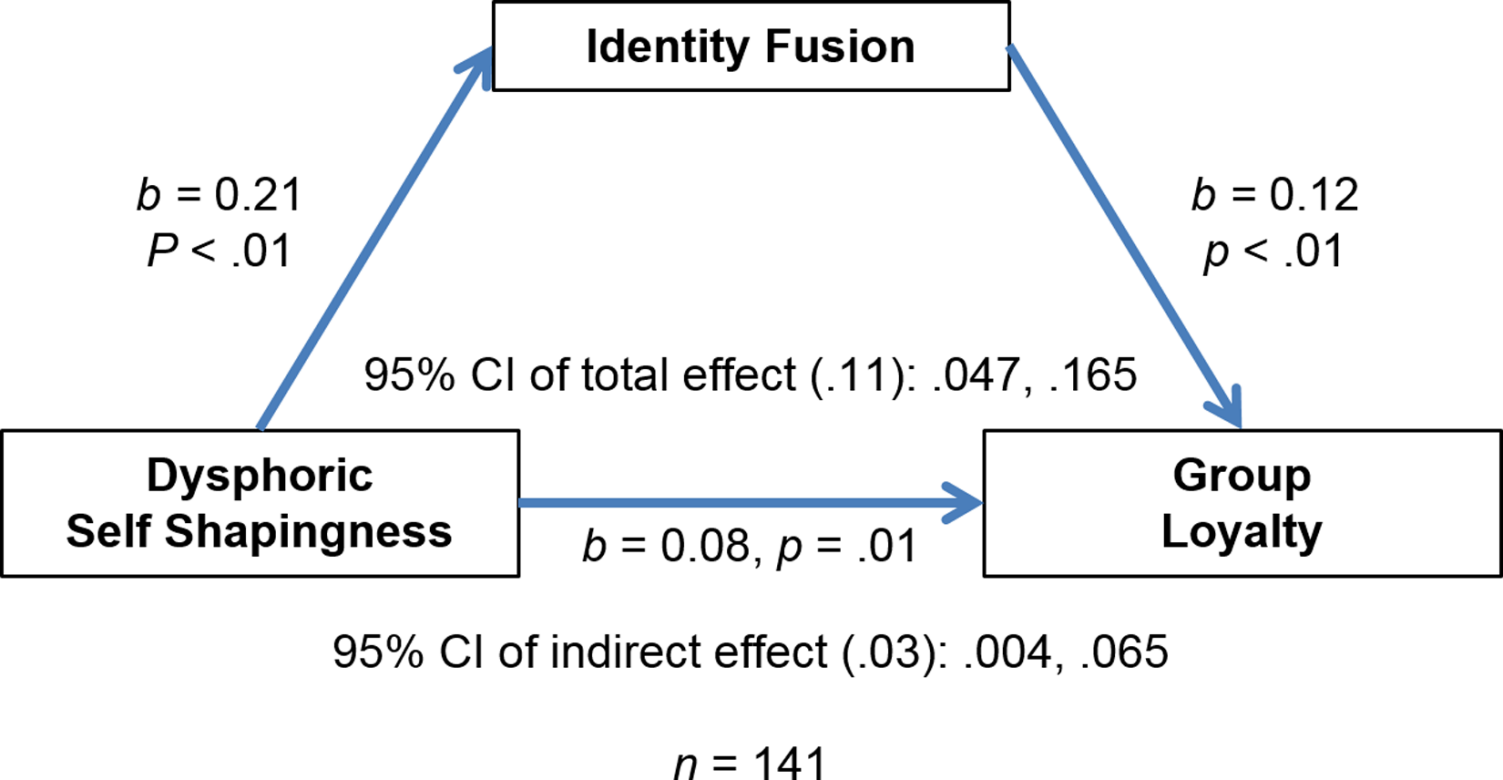
Mediation analysis shows that identify fusion mediates the relationship between dysphoric self-shapingness and group loyalty.

In model 2 the outcome was loyalty, fusion the mediator, dysphoric self-shapingness the predictor, and past investment a covariate (Fig 3). As seen in Table 7, the confidence intervals for the indirect effects did not cross zero. There was evidence of a direct effect of dysphoric self-shapingness on loyalty, independent of past investment and fusion. In sum, these two models supported the hypothesis that both euphoric and dysphoric events induce self-shapingness that leads to fusion. Furthermore, there was evidence to suggest that dysphoric events act on the individual to increase loyalty independently of past investment. We re-ran both models to control for identification and found our results to be robust for dysphoric self-shapingness (*z* = 1.89, *p* = .06) and euphoric self-shapingness (*z* = 2.00, *p* = .05).

**Table 7:** Total, direct, and indirect effects for dysphoric self-shapingness predicting loyalty with fusion as a predictor and past investment as a covariate.

	Effect	SE	95% CI
Total effect	.11	.03	.047, .165
Direct effect	.08	.03	.021, .140
Indirect effect	.03	.02	.003, .065

## Discussion

Our results indicate that identity fusion provides a novel explanation of group loyalty, independent from the effects of identification and cognitive dissonance. In support of Whitehouse & Lanman’s earlier assertion (2014), we provide the first evidence that identity fusion is not simply a by-product of cognitive dissonance. We also found evidence to support the claim that identity fusion arises once an individual has experienced group events that they believe to be personally self-shaping. In addition, we found that euphoric and dysphoric events, when seen as highly self-shaping, were both likely to result in strong levels of fusion and lifelong loyalty.

This study provides further evidence that certain group events (e.g., key group victories and defeats) have the power to strongly shape personal identity and the relationship between personal and social identities [10, 24]. In contrast to mechanisms that cut off reflected failure or bask in reflected glory associated with more fluid forms of group alignment such as identification [37–39], once people become fused they exhibit an unerring group loyalty [7, 16]. The extreme pro-group activities that fused individuals engage in are also well documented [9, 11, 12, 13, 14]. Such acts of group commitment can be harnessed by commercial enterprises (e.g., football clubs) and the socio-political sphere (e.g. military groups); and for both good and bad outcomes. Be it businesses profiteering from creating ‘shaping’ events for sports fans in the billion pound sports and leisure industry, or recognising the importance of terrorist attacks in fusing citizens to nations or insurgents to terrorist groups—understanding the mechanisms through which people become fused to a group entails significant implications at a societal level.

Our results also support and extend understanding of the role of cognitive dissonance in relation to lifelong loyalty. One’s past time invested in supporting the ingroup represents an extension of the free-choice paradigm, whereby the individual’s past choices affect their future behaviours and beliefs [33, 40]. Our results suggested that greater experiences of dissonance can lead to increases in loyalty, but we also provided novel evidence for identity fusion’s role in lifelong loyalty, independent of a traditional dissonance account. Dissonance has been unable to answer some questions that fusion seems more able to explain. For instance, it seems far-fetched to think that group members would engage in extremely costly pro-group acts–even making the ultimate sacrifice of their own lives [12]–in order to reduce the aversive experience of cognitive dissonance. Instead, identity fusion theory details how such acts arise from intense shared group events that transform the personal and social self, leading one to feel a unique responsibility to defend one’s psychological kin at any cost.

Our results specifically identified self-shaping group victories and defeats as key to the relationship between fusion and lifelong loyalty, which supports the current theory that one’s personal and social selves can ‘fuse’ together resulting in an extraordinary pro-group mentality [20, 21, 26, 41]. Unpacking the cognitive nuances of lifelong loyalty and extreme group commitment is a delicate operation and, while traditional dissonance accounts may partially explain the phenomenon of lifelong loyalty, our results suggest that the theory of identity fusion and its associated mechanisms provide a fuller explanation.

Our study is not without limitations. First, this study was correlational in nature and, though our proposed direction of causality is supported by theory, we cannot be resolutely decided on this. With this study as a basis, future experimental work could address the causality issue. Second, this study used self-reports, which are potentially marred by problems of subjectivity, bias, and deliberate deceit [42, 43]. Self-reports also focus on participants’ explicit feelings, rather than the implicit drives we might be most interested in for this kind of research. Indeed, a substantial body of literature suggests that the unconscious mind drives much of our thoughts, feelings and behaviours [44–46].

As such, the self-reporting issue leaves this study open to the criticism that unconscious processes could be motivating a sense of self-shapingness following a dysphoric event (e.g. to reduce dissonance), when the individual hasn’t actually been transformed in any empirically discernible fashion. Such a claim would be hard to demonstrate scientifically, but future research should aim to investigate such claims by accessing participants’ unconscious responses, perhaps using implicit association tasks. Nevertheless, self-report measures are quick and simple to administer and are thus used regularly in psychological research. In this exploratory study, our self-report measures of self-shapingness were brief and future research will need to explore issues of construct validity in more detail.

Third, we propose the development of two measures for inclusion in future related projects. The relationship between self-shapingness and reflection (Jong et al., 2015) is left unexplored in this study, a relationship that we imagine will be fruitful to researchers investigating the antecedents of identity fusion. Measures of affective intensity in relation to significant events were also omitted from this study and future research could explore ways of appropriately testing affect post-event. By improving our understanding of the affective processes underlying the perceptions of self-shapingness, we stand a better chance of comprehending how self-shapingness–one of the first proposed mediators of identity fusion–occurs.

Finally, and perhaps most importantly, are limitations associated with a convenience sample. While the variability of football clubs sampled, age range, and mix of educational background goes some way to achieving a representative sample, issues of selection and social acceptability biases cannot be dismissed. Further research would need to be conducted, ideally with a new population, to confirm the generalisability of our claim that a sense of being shaped by intense shared events, including dysphoric self-shapingness, mediates the relationship between fusion and lifelong loyalty independently of cognitive dissonance.

Research into identity fusion and its antecedents offers us unique opportunities to understand some of the most extreme social behaviours in our species and has the potential to help us channel the pro-group sentiments of fused individuals for the good. For instance, research could tell us if individuals who have been shaped by trauma and despair are more inclined to act with hostility to out-groups, perceiving out-groups as threats based on their self-shaping memories, or whether euphoric self-shaping experiences foster acts of extraordinary self-sacrifice and acts of charity. By deconstructing the path(s) to fusion and understanding each stepping stone in the process, research can help develop cognitive and therapeutic approaches to de-fusion in order to realign individuals away from groups that pose societal risks. Such research may thus provide a practical tool for national security–be it curbing local football hooliganism or fighting the global ‘war on terror’. The possibility of creating de-fusion programmes opens up an ethical minefield and the development of any such initiatives would need thorough research in place to support them, as well as wide discussion involving all potential stakeholders. With the knowledge that dysphoric events contribute greatly to fusion, we are also in a position to advocate a reduction in the ill treatment of marginal groups; for these are the experiences likely to hold together disenfranchised collectives built on values of self-preservation and out-group hostility.

## Supporting Information

S1 File Dataset for ‘lifelong loyalty’ in football fans. (SAV)

S2 File Additional analyses. Analyses including the duration of support and age variables.
(DOCX)
