# Supplementary material for: Explaining Lifelong Loyalty: The Role of Identity Fusion and Self-Shaping Group Events
Source: PLoS One. 2016 Aug 10;11(8):e0160427. doi: 10.1371/journal.pone.0160427 (PMC4980014; doi:10.1371/journal.pone.0160427)
Supplement: S2 File — Analyses including the duration of support and age variables. (DOCX) [file pone.0160427.s002.docx]

**Supplementary file 1**

**Age vs. raw years of support vs. duration of support (%)**

Duration of support was calculated by making years of support a percentage of the participant’s age. We repeated analyses including the two raw variables (age and years of support) and found that these analyses were similar to the single ‘duration of support’ variable.

Descriptives and correlations for all variables, including age and raw years of support are reported in Table 1a.

*Table 1a: Descriptive statistics and correlations for identity fusion, identification, loyalty, self-shaping events, and past investment*

|  | *Self-shap.* | *Identific.* | *Investment*  *(duration of support %)* | *Fusion* | *Loyalty* | ***Age*** | ***Years of support*** |
| --- | --- | --- | --- | --- | --- | --- | --- |
| *Self-shap.* | *3.67 (1.68)* |  |  |  |  | *.02 (.84)* | *.14 (.10)* |
| *Identific.* | *.13 (.11)* | *5.17 (1.52)* |  |  |  | *.05 (.53)* | *.04 (.67)* |
| *Investment* | *.19 (.04)** | *-.003 (.97)* | *68.81 (24.72)* |  |  | *.17 (.04)** | *.74 (<.01)** |
| *Fusion* | *.40 (<.01)** | *.57 (<.01)** | *.09 (.28)* | *3.88 (1.38)* |  | *-.01 (.89)* | *.03 (.71)* |
| *Loyalty* | *.34 (<.01)** | *.16 (.06)** | *.44 (<.01)** | *.32 (<.01)** | *6.48 (.73)* | *-.02 (.85)* | *.29 (<.01)** |
| ***Age*** |  |  |  |  |  | *37.51 (13.33)* |  |
| ***Years of support*** |  |  |  |  |  | *.74 (<.01)** | *26.36 (14.08)* |

*Note: Means and SD (in parentheses) on the diagonal, Pearson’s r’s and p-values (in parentheses) below the diagonal. A * denotes significance.*

Hypothesis 1: Identification, dissonance, and fusion predict loyalty, but only the effects of dissonance and fusion hold in a simultaneous regression.

We re-ran the regression analyses but this time entered age and years of support instead of duration of support (%) to test past investment (Table 2a). The effects were similar to our compound variable.

*Table 2a: Linear regression with identification, age, years of support, and fusion entered as variables to predict group loyalty.*

| Model | | Unstandardized Coefficients | | Standardized Coefficients | t | Sig. (*p*) |
| --- | --- | --- | --- | --- | --- | --- |
|  |  | B | Std. Error | Beta |  |  |
| 1 | (Constant) | 6.07 | .22 |  | 27.56 | <.01 |
|  | Identification | .08 | .04 | .16 | 1.94 | .06 |
| 2 | (Constant) | 6.12 | .28 |  | 22.24 | <.01 |
|  | Identification | .08 | .04 | .16 | 1.94 | .05 |
|  | Age | -.001 | .01 | -.02 | -.27 | .79 |
| 3 | (Constant) | 6.22 | .25 |  | 25.23 | <.01 |
|  | Identification | .08 | .04 | .16 | 2.15 | .03 |
|  | Age | -.03 | .01 | -.51 | -4.59 | <.01 |
|  | Years of support | .03 | .01 | .66 | 5.96 | <.01 |
| 4 | (Constant) | 6.01 | .25 |  | 24.47 | <.01 |
|  | Identification | -.003 | .04 | -.01 | -.07 | .95 |
|  | Age | -.03 | .01 | -.48 | -4.46 | <.01 |
|  | Years of support | .03 | .01 | .63 | 5.91 | <.01 |
|  | Mean fusion to club | .16 | .05 | .30 | 3.40 | <.01 |
| a. Dependent Variable: Loyalty | | | | | | |

Model 1: *R^2^* = .03, *F(139)* = 3.75*, p* = .06

Model 2: *R^2^* = .03, *F(138)* = 1.90*, p* = .15

Model 3: *R^2^* = .23, *F(137)* = 13.42*, p* < .001

Model 4: *R^2^* = .29, *F(136)* = 13.73*, p* < .001

Hypothesis 2: The path to fusion is self-shapingness, not dissonance.

We re-ran Process (Model 4) twice more, where the outcome was loyalty, fusion the mediator, self-shapingness the predictor, and age (Sobel *z* = 2.28, p=.02, Table 3a) or years of support (Sobel *z* = 2.40, p=.02, Table 3b) as covariates. The effects were similar to our compound variable.

*Table 3a: Total, direct, and indirect effects for self-shapingness predicting loyalty (outcome) via fusion and age as a covariate*

|  | Effect | SE | 95% CI |
| --- | --- | --- | --- |
| Total effect | .14 | .03 | .080, .217 |
| Direct effect | .11 | .04 | .036, .183 |
| Indirect effect | .04 | .02 | .005, .116 |

*Table 3b: Total, direct, and indirect effects for self-shapingness predicting loyalty (outcome) via fusion and years of support as a covariate*

|  | Effect | SE | 95% CI |
| --- | --- | --- | --- |
| Total effect | .13 | .03 | .066, .200 |
| Direct effect | .09 | .04 | .022, .165 |
| Indirect effect | .04 | .02 | .004, .090 |

Hypothesis 3: Self-shapingness following both euphoric and dysphoric events predicts fusion.

We re-ran Model 4 four more times: for euphoric self-shapingness with age as a covariate (Sobel *z* = 2.34, p=.02, Table 4a) and years of support as a covariate (Sobel *z* = 2.49, p=.01, Table 4b); then for dysphoric self-shapingness with age as a covariate (Sobel *z* = 2.24, p=.03, Table 4c) and years of support as a covariate (Sobel *z* = 2.28, p=.02), Table 4d). The effects were similar to our compound variable.

*Table 4a: Total, direct, and indirect effects for euphoric self-shapingness predicting loyalty (outcome) with fusion as a predictor and past investment as a covariate*

|  | Effect | SE | 95% CI |
| --- | --- | --- | --- |
| Total effect | .12 | .03 | .059, .185 |
| Direct effect | .08 | .04 | .011, .150 |
| Indirect effect | .04 | .02 | .004, .093 |

*Table 4b: Total, direct, and indirect effects for euphoric self-shapingness predicting loyalty (outcome) with fusion as a predictor and years of support as a covariate*

|  | Effect | SE | 95% CI |
| --- | --- | --- | --- |
| Total effect | .10 | .03 | .048, .170 |
| Direct effect | .07 | .03 | -.001, .133 |
| Indirect effect | .04 | .02 | .003, .096 |

*Table 4c: Total, direct, and indirect effects for dysphoric self-shapingness predicting loyalty with fusion as a predictor and age as a covariate*

|  | Effect | SE | 95% CI |
| --- | --- | --- | --- |
| Total effect | .13 | .03 | .064, .193 |
| Direct effect | .10 | .03 | .035, .165 |
| Indirect effect | .03 | .02 | .006, .068 |

*Table 4d: Total, direct, and indirect effects for dysphoric self-shapingness predicting loyalty with fusion as a predictor and years of support as a covariate*

|  | Effect | SE | 95% CI |
| --- | --- | --- | --- |
| Total effect | .12 | .03 | .053, .178 |
| Direct effect | .09 | .03 | .024, .150 |
| Indirect effect | .03 | .02 | .005, .071 |
